# Durable response to Olaparib in EGFR and somatic BRCA2-mutated lung adenocarcinoma with leptomeningeal metastases: a case report

**DOI:** 10.3389/fonc.2025.1512886

**Published:** 2025-03-20

**Authors:** Fang-fang Hou, Li-rong Liu, Wen-jie Zhao, Yan-chun Qu, Rui Zhou, Tian-lin Wang, Yong-song Ye, Xiao-shu Chai, Hai-bo Zhang

**Affiliations:** ^1^ The Second Clinical College of Guangzhou University of Chinese Medicine, Guangzhou, China; ^2^ Department of Oncology, The Second Affiliated Hospital of Guangzhou University of Chinese Medicine, Guangdong Provincial Hospital of Traditional Chinese Medicine, Guangzhou, China; ^3^ Guangdong-Hong Kong-Macau Joint Laboratory on Chinese Medicine and Immune Disease Research, The Second Affiliated Hospital of Guangzhou University of Chinese Medicine, Guangzhou, China; ^4^ Guangdong Provincial Key Laboratory of Clinical Research on Traditional Chinese Medicine Syndrome, The Second Affiliated Hospital of Guangzhou University of Chinese Medicine, Guangzhou, China

**Keywords:** somatic BRCA2 mutation, Olaparib, leptomeningeal metastases, non-small cell lung cancer, case report

## Abstract

Mutations in breast cancer susceptibility genes 1/2 (BRCA1/2) are strongly associated with a significantly higher risk of numerous cancers, including ovarian, breast, prostate, and pancreatic cancer. PARP inhibitors have been approved for the treatment of ovarian and breast cancer. However, studies focusing on the association between the BRCA gene and NSCLC, as well as the efficacy of PARP inhibitors in NSCLC, are scarce. Here, we present the case of a patient with lung adenocarcinoma harboring EGFR and somatic BRCA2 mutations, who developed resistance to third-generation EGFR tyrosine kinase inhibitors (TKIs) and subsequently exhibited durable response to Olaparib. This case exemplifies the remarkable efficacy of precision-targeted therapy in combination with intrathecal chemotherapy, which has resulted in significant clinical improvement for an EGFR- and BRCA-mutant lung cancer patient suffering from severe and symptomatic leptomeningeal metastases. Our findings provide clinical evidence and guidance for the treatment of NSCLC patients with BRCA mutations. Nonetheless, further studies are warranted to elucidate the role of BRCA mutations in NSCLC.

## Introduction

Leptomeningeal metastases (LM) are one of the most prevalent complications in patients with advanced non-small-cell lung cancer (NSCLC), with a prevalence ranging from 3% to 5% (1, 2). Notably, the morbidity increases to 9% in epidermal growth factor receptor-mutated (EGFRm) NSCLC patients (2, 3). Patients with leptomeningeal metastases typically have a poor prognosis. Due to advances in immunotherapy and targeted therapy for NSCLC, the median survival has been prolonged to 3-11 months (4). However, treatment options remain limited for patients with drug resistance.

The breast cancer susceptibility genes (BRCA) are tumor suppressor genes including BRCA1 and BRCA2. Through the homologous recombination repair (HRR) pathway, BRCA proteins participate in the DNA double-strand break repair (5). BRCA mutations resulting in homologous recombination deficiency (HRD) are associated with a high risk of cancers, such as ovarian, breast, and pancreatic cancer. According to The Cancer Genome Atlas database, BRCA1 and BRCA2 variants were observed in 3.0% and 4.8% of lung adenocarcinoma patients, respectively (6). The prevalence of germline BRCA mutation was 1.3% in advanced Chinese NSCLC patients (7).

Poly (ADP-ribose) polymerase (PARP) is a key ribozyme participating in repairing the DNA single-strand breaks. Conversely, PARP inhibitors prevent the repair of DNA single-strand breaks, leading to the accumulation of DNA damage and cell death through synthetic lethality for tumors displaying HRD. Olaparib, an oral PARP inhibitor exhibiting high clinical efficacy in patients with germline BRCA-mutated malignancies, is approved for the treatment of breast and ovarian cancers. However, the role of BRCA mutation and the efficacy of PARP inhibitors in NSCLC remains elusive.

This article outlined the case of a NSCLC patient with brain and leptomeningeal metastases, harboring EGFR and somatic BRCA2 mutations, who developed resistance to the third-generation EGFR-TKIs but nonetheless demonstrated a lasting positive response to Olaparib.

## Case presentation

A 67-year-old female with no smoking history and no significant medical history presented to the local hospital with a two-month history of delayed communication responses. Her family history included two sisters were diagnosed with breast cancer, but further details were unavailable. ^18^F-FDG PET/CT revealed a nodule in the lower lobe of the right lung, and the patient was diagnosed with lung cancer. Lymph nodes in the right hilum and mediastinum with hypermetabolism were indicative of metastases. Brain MRI displayed multiple brain metastatic lesions in the left frontal lobe, parietal lobe, right basal ganglia, left temporal lobe, and cerebellar hemisphere. She underwent left frontal mass resection and postoperative pathological examination revealed moderately to poorly differentiated adenocarcinoma, with immunohistochemistry (IHC) staining confirming adenocarcinoma of lung origin. Lastly, the tissue-based next-generation sequencing (NGS) identified 13 genomic alterations, including an L858R mutation in exon 21 of EGFR and somatic mutation in BRCA2 p. L2368Ffs*24 as well as mutations in FANCC, RBM10, CCND2, IGF1R, TSC2, ALK, HDAC4, and HIST1H1C (**Table 1**).

**Table 1:** Genomic alterations detected by next-generation sequencing (tissue specimens obtained from brain metastases in the left frontal lobe in February 2021).

Gene	Transcript	c.HGVS	p.HGVS	Variant allele frequency (%)
EGFR	NM_005228.3	c.2573T > G	p.L858R	32.2
BRCA2	NM_000059.3	c.7103_7104insT	p.L2368Ffs*24	25.3
RBM10	NM_001204468.1	c.2543T > G	p.L848R	42.5
RBM10	NM_001204468.1	c.2550+1G > T	–	40.0
FANCC	NM_000136.2	c.287G > T	p.C96F	30.5
CCND2	NM_001759.3	c.755C > T	p.A252V	27.1
IGF1R	NM_000875.3	c.639A > T	p.K213N	25.6
TSC2	NM_000548.3	c.2689T > C	p.F897L	20.8
ALK	NM_004304.4	c.356A > T	p.E119V	19.8
HDAC4	NM_006037.3	c.2266-12_2268delTTGTTTCCACAGGTG	--	16.0
HIST1H1C	NM_005319.3	c.528_551delGGCCAAGGTTGCGAAGCCCAAGAA	p.K178_A185del	8.1
RECQL4	NM_004260.3	amplification		CN: 4.8
MYC	NM_002467.4	amplification		CN: 3.8

HGVS, Human Genome Variation Society; CN, Copy number.

**Figure 1:**
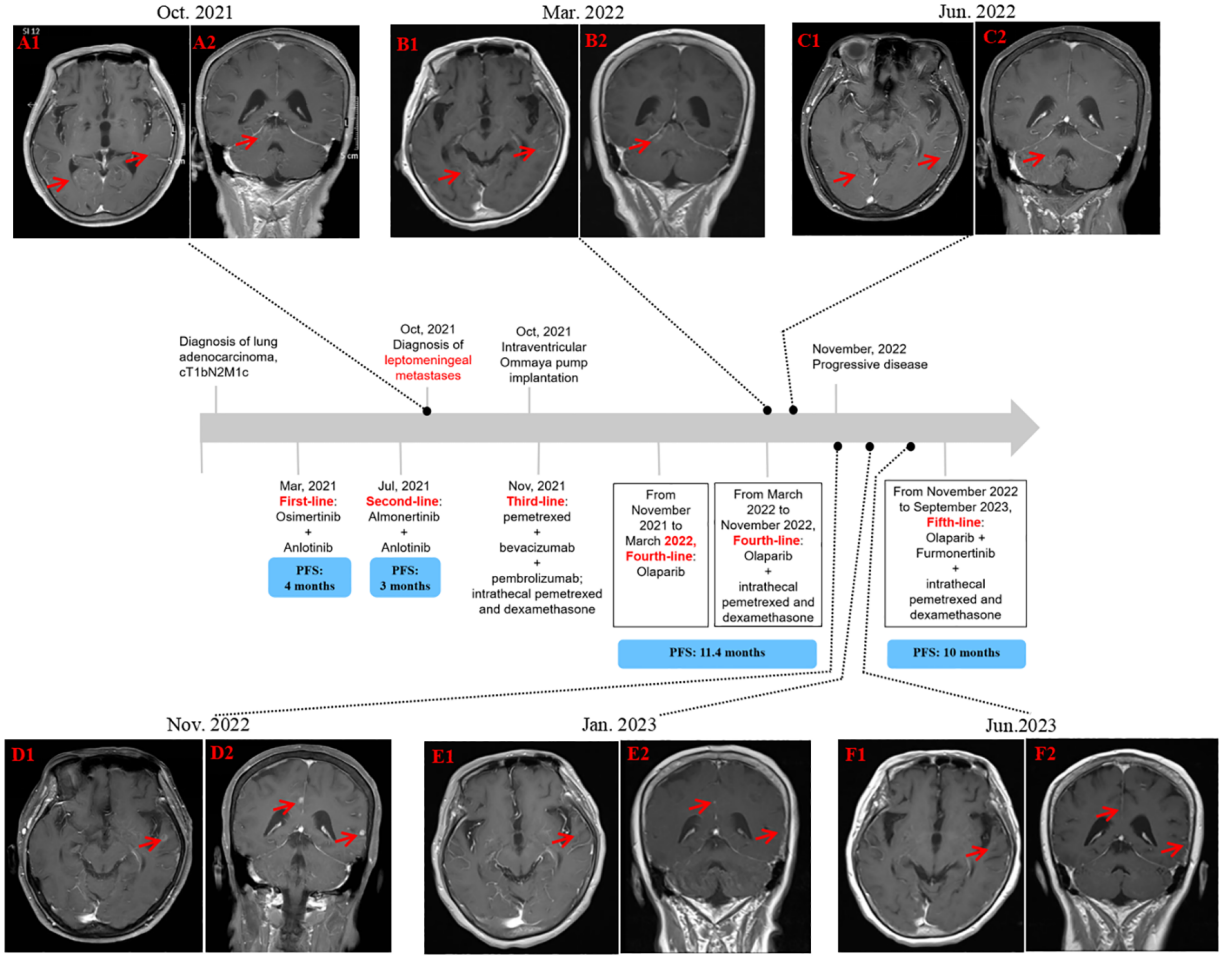
Schematic representation of the diagnostic and treatment process. **(A1, A2)** MRI illustrating leptomeningeal metastases. **(B1, B2)** MRI depicting a decrease of leptomeningeal metastases after 3 months of Olaparib. **(C1, C2)** MRI displaying a continuous reduction in the degree of leptomeningeal metastases after 6 months of Olaparib in combination with 2 cycles of intrathecal pemetrexed. **(D1, D2)** The number of metastatic brain tumors increased, with a PFS of 11.4 months. **(E1, E2)** MRI delineating partial response after 2 months of Olaparib and Furmonertinib in combination with 2 cycles of intrathecal pemetrexed. **(F1, F2)** MRI reflecting continuous response following the intake of Olaparib and Furmonertinib for approximately 8 months. MRI, magnetic resonance imaging.

Osimertinib (80mg oral administration daily) in combination with Anlotinib (12mg, d1-14, q3w) was initiated as the first-line treatment. However, there was no relief in her symptoms, and the treatment efficacy was stable while she experienced some side effects, such as diarrhea, due to the treatment. She was subsequently switched to Almonertinib (110mg oral administration daily) plus Anlotinib (12mg, d1-14, q3w) three months later. After treatment for an additional 3 months, the patient complained of increased fatigue, blurred vision, nausea, vomiting, occasional headache, and dizziness. The brain MRI (**Figure 1A**) showed multiple dot-line enhancements in the cerebral and cerebellar sulci, indicating the presence of leptomeningeal metastases. A lumbar puncture was performed in our department, and adenocarcinoma cells were identified in the cerebrospinal fluid (CSF) through cytological examination.

Intraventricular Ommaya pump implantation for leptomeningeal metastases was conducted. Afterward, CSF genetic testing was conducted to identify the mechanisms of resistance, exposing mutations in EGFR p.L858R and BRCA2 p.L2368Ffs*24, accompanied by TET2 and TSC2 (**Table 2**). The third line treatment, pemetrexed (500 mg/m^2^ intravenously on day 1 every 3 weeks) plus bevacizumab (7.5 mg/kg intravenously on day 1 every 3 weeks) and pembrolizumab (200mg intravenously on day 1 every 3 weeks) were administrated while pemetrexed combined with dexamethasone was delivered via the Ommaya reservoir. However, the regimen was terminated 5 days after the first cycle because of severe bone marrow suppression and serious infection resulting from chemotherapy. Through interventions such as anti-infection, promoting leucocytes and thrombocyte, and blood transfusion, the patient was successfully rescued. Unfortunately, symptoms of leptomeningeal metastases worsened dramatically. She was lethargic, unresponsive, experienced difficulty in eating and swallowing, and necessitated intravenous nutritional support. Her condition rapidly deteriorate in the following days, with her Eastern Oncology Cooperative Group (ECOG) performance status (PS) declining to 4.

**Table 2:** Genomic alterations of CSF ctDNA detected by next-generation sequencing.

CSF ctDNA Assay October 25, 2021			CSF ctDNA Assay November 14, 2022			CSF ctDNA Assay June 28, 2023		
Gene	p.HGVS	AF(%)	Gene	p.HGVS	AF(%)	Gene	p.HGVS	AF(%)
EGFR	p.L858R	9.7	EGFR	p.L858R	35.4	EGFR	p.L858R	5.2
BRCA2	p.L2368Ffs*24	6.3	BRCA2	p.L2368Ffs*24	11.6	BRCA2	p.L2368Ffs*24	9.0
TSC2	p.F897L	5.0	TSC2	p.F897L	12.0	TSC2	p.F897L	4.6
TET2	p.I1873T	0.2	CDKN2A	Rearrangement	46.0	CDKN2A	Rearrangement	33.0
						RBM10 c.2355+1G>T	–	14.1
						FGFR1	p.R68Q	9.1
						CCND2	p.A252V	15.4
						RBM10	p.L783R	15.3

AF, allele frequency; ctDNA, circulating tumor DNA; HGVS, Human Genome Variation Society.

**Figure 2:**
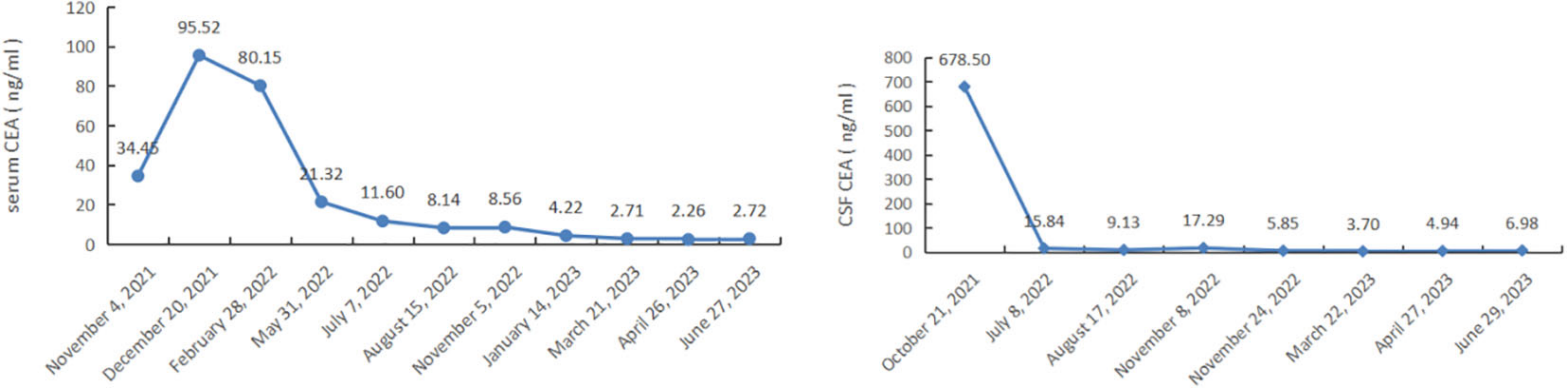
Fluctuation of CEA (normal value: 0-5 ug/L) levels in the CSF and blood.

Based on the presence of a somatic mutation in BRCA2, she was treated with oral Olaparib as the forth line treatment. Because of dysphagia, the patient initially only received 150mg of Olaparib once a day. Due to her poor initial condition and inability to tolerate chemotherapy, intrathecal chemotherapy via the Ommaya reservoir was not initiated until March 2023. Her condition improved after 10 days on Olaparib. After one month, Olaparib was gradually increased to 300 mg twice a day. Her leptomeningeal metastases-related symptoms relieved remarkably. Her ECOG performance status improved to 2, while CSF carcinoembryonic antigen (CEA) levels similarly decreased (**Figure 2**). She regained normal eating habits, mobility, and self-care abilities. Indeed, her quality of life was significantly improved. At the three-month follow-up, computed tomography showed a stable disease of the primary tumor in lung, while brain MRI (**Figure 1B**) revealed a reduction in leptomeningeal metastases. After 6 months of Olaparib, there was a significant decrease in leptomeningeal metastases on brain MRI (**Figure 1C**), and the primary lung cancer remained well-controlled since the diagnosis. The only adverse event reported was grade 3 bone marrow suppression. Ultimately, the patient achieved a progression-free survival of 11.7 months, which was longer than the first line of Osimertinib/Almonertinib plus Anlotinib.

In November 2022, MRI images (**Figure 1D**) illustrated progression in brain and leptomeningeal lesions, while the CT indicated a stable disease for extracranial lesions. Another Genomic profiling of CSF through the next-generation sequencing (Illumina NovaSeq 6000) technology identified a higher frequency of mutations for EGFR L858R and sBRCA2 p.L2368Ffs*24 mutations (**Table 2**). Subsequently, Furmonertinib, an irreversible and selective third-generation EGFR tyrosine kinase inhibitor, was administered at a dose of 160 mg once daily in combination with Olaparib (300 mg once daily) as the fifth-line treatment. The patient showed a partial response for brain metastases after 2 months (**Figure 1E**). As of this writing, the patient has been on Furmonertinib and Olaparib for 7 months and maintains a good quality of life. After the initial diagnosis of leptomeningeal metastases in October 2021 to the data cutoff for this case report, she has been taking Olaparib for over 18 months, with a continuous response noted in June 2023 (**Figure 1F**). A schematic of her clinical history and lines of treatment is displayed in **Figure 1**.

## Discussion

To the best of our knowledge, this is the first study to successfully apply Olaparib monotherapy or in combination with Furmonertinib and intrathecal chemotherapy for NSCLC patients with EGFR and somatic BRCA2 co-mutation following resistance to third-generation EGFR-TKIs. Our patient, who harbored both EGFR and somatic BRCA2 mutations, demonstrated a durable response to Olaparib lasting approximately 21 months, representing the longest reported duration of response to Olaparib in NSCLC with LM to date. This surpasses previous case reports (8, 9), where the duration of Olaparib response was 13.5 months and 8 months, respectively. The patient experienced initial monotherapy with Olaparib, followed by combination therapy with intrathecal chemotherapy, and eventually a triple combination of Olaparib, Furmonertinib, and intrathecal chemotherapy. This sequential approach highlights the potential of PARP inhibitors in managing EGFR and BRCA co-mutated NSCLC with LM.

Currently, there is no standard of care for the treatment of non-small cell lung cancer patients with leptomeningeal metastases. Once patients progress on TKIs or platinum-based chemotherapy, there is a lack of effective treatment options. Systemic therapy, including targeted therapy and chemotherapy, combined with local treatment, such as radiotherapy and intrathecal chemotherapy, are the most common therapy for these patients. There is still no consensus on when the optimal time is to implement whole-brain radiotherapy and whether it is a beneficial treatment for patients with leptomeningeal metastases from NSCLC. When the brain MRI suggested leptomeningeal metastases and the patient presented with symptoms of intracranial hypertension such as nausea and vomiting, lumbar puncture was quickly performed by our medical team. As the cerebrospinal fluid cytology confirmed leptomeningeal metastases, an Ommaya reservoir was subsequently installed for the patient.

The Ommaya reservoir is a preferred approach for intrathecal chemotherapy. Additionally, it facilitates CSF drainage to alleviate symptoms of intracranial hypertension and enables CSF collection for detecting tumor marker levels and CSF ctDNA, which are crucial for assessing treatment efficacy and prognosis. What’s more, intrathecal chemotherapy via Ommaya reservoirs is more convenient and safer than lumbar puncture. In a phase I/II clinical trial, intrathecal pemetrexed has been shown to be a potential treatment for patients with EGFR-mutant NSCLC-LM resistance to TKIs (10). Given the poor prognosis of the patients with extensive leptomeningeal metastases and resistance to EGFR-TKIs, intrathecal pemetrexed 50mg every 4 weeks was administrated. When combined with Olaparib, the dose of pemetrexed was reduced to 30mg after two cycles because of grade three myelosuppression.

The patient’s poor response to Osimertinib and Almonertinib initially, followed by a significant clinical improvement upon initiation of Olaparib monotherapy, suggests that the somatic BRCA2 mutation may have acted as a co-driver alongside EGFR, contributing to tumor progression and resistance to EGFR-TKIs. This observation underscores the potential role of BRCA2 mutations in NSCLC. However, the impact of BRCA2 mutations, particularly somatic versus germline variants, on the efficacy of targeted therapies in NSCLC remains poorly understood. While some studies suggest that germline BRCA mutations may confer sensitivity to platinum-based chemotherapy, the influence of somatic BRCA2 mutations on EGFR-TKI efficacy is less clear. Notably, a previous study (7) reported a trend toward improved overall survival in EGFR-mutant NSCLC patients with concurrent germline BRCA mutations treated with EGFR-TKIs, though this finding was not statistically significant. In contrast, retrospective data from our institution indicates that NSCLC patients with BRCA mutations may exhibit poorer responses to first-line EGFR-TKIs. This discrepancy highlights the need for further research to elucidate the role of BRCA mutations in NSCLC and their impact on targeted therapy outcomes. A pre-clinical study demonstrated that in EGFR mutant lung cancer cells, TKI-resistant cells exhibit sensitivity to PARP inhibitors, potentially mediated by NOX-dependent reactive oxygen species (11). Based on this, the researchers conducted a phase I clinical trial combining Niraparib with Osimertinib in TKI-resistant EGFR mutant NSCLC (NCT03891615). However, the trial did not report any results. Simultaneously, a similar phase II study reported no statistically significant difference in PFS between Olaparib in combination with gefitinib and gefitinib monotherapy as first-line therapy for patients with EGFR-mutant NSCLC (12). We speculated that the negative outcomes may be attributed to inadequate selection of patients based on their BRCA status in the study. A preclinical study indicated that knockdown of BRCA1 or BRCA2 led to increased sensitivity of A594 cells to Olaparib, compared with the wild type control cells (13). This study further indicates that pathogenic BRCA2 mutations could serve as a biomarker for predicting the efficacy of PARP inhibitors in lung cancer patients. Nevertheless, I believe it is meaningful to conduct prospective clinical trails to evaluate the efficacy and prognosis of EGFR-TKIs for NSCLC patients currents with BRCA mutations.

In addition to the BRCA2 mutation, our patient harbored co-occurring mutations in FANCC, RBM10, TET2, and TSC2, which may have further contributed to the resistance to EGFR-TKIs. While the functional significance of these mutations in NSCLC remains unclear, they likely played a role in the patient’s poor response to Osimertinib and Almonertinib. Unlike well-characterized resistance mechanisms such as TP53 mutations or MET amplification, the impact of TET2 and TSC2 mutations on EGFR-TKI efficacy is not well established. This case highlights the complexity of genetic alterations in NSCLC and their potential influence on treatment outcomes.

The patient’s failure to respond to systemic chemotherapy may be attributed to the rapid progression of LM, low cerebrospinal fluid (CSF) penetration of chemotherapeutic agents, and poor tolerance to intensive chemotherapy regimens. The mechanisms underlying BRCA2-mediated resistance to EGFR-TKIs remain unknown and warrant further investigation. Similarly, the eventual resistance to the combination of Furmonertinib and Olaparib is not fully understood. Despite dynamic monitoring of CSF ctDNA, we were unable to identify specific resistance mechanisms, underscoring the need for more comprehensive genomic analyses in future studies.

This case highlights the crucial significance of CSF genetic testing in patients with leptomeningeal metastases, particularly when intracranial progression occurs despite stable extracranial disease. The detection of ctDNA in CSF provides a valuable supplement to conventional cytopathology, enabling more accurate diagnosis and monitoring of treatment responses (14). Our results further corroborated that the mutation frequency of EGFR-mutated DNA substantially decreased with effective treatment (15). Moreover, CSF circulating tumor DNA (ctDNA) outperformed plasma ctDNA in determining the molecular characteristics of NSCLC patients with leptomeningeal metastases and may aid in identifying the mechanism underlying drug resistance and guiding treatment (16). Taken together, CSF liquid biopsy is a promising approach for the diagnosis of leptomeningeal metastases, the detection of genetic variants, and the monitoring treatment responses.

In conclusion, we report a refractory lung adenocarcinoma patient with extensive leptomeningeal metastases, harboring EGFR and somatic BRCA2 co-mutations, who exhibited a lasting response to Olaparib despite resistance to third-generation EGFR-TKIs. This case represents one of the longest reported durations of response to Olaparib in an EGFR and BRCA2 co-mutated NSCLC patient with leptomeningeal metastases, underscoring the potential of PARP inhibitors in combination with targeted therapy and intrathecal chemotherapy for managing this challenging patient population. However, the role of BRCA mutations in NSCLC and their impact on treatment outcomes remain controversial, necessitating further research to optimize therapeutic strategies for this subset of patients. Our findings provide valuable insights into the management of NSCLC with co-occurring EGFR and BRCA mutations and highlight the importance of precision medicine in overcoming treatment resistance.

## Data Availability

The datasets presented in this study can be found in online repositories. The names of the repository/repositories and accession number(s) can be found in the article/**Supplementary Material**.
